# Coalmining and the National Scheme for Disabled Ex-Servicemen after the First World War

**DOI:** 10.1080/03071022.2016.1144311

**Published:** 2016-03-31

**Authors:** Mike Mantin

**Affiliations:** ^a^ Swansea University

**Keywords:** Coalmining, disability, employment, First World War, King’s Roll

## Abstract

After the First World War, disabled British veterans returned home to an uncertain future of work. In addition to voluntary efforts, the government’s response to the national employment crisis – the National Scheme for Disabled Ex-Servicemen (commonly known as the King’s Roll) – was established in 1919 to encourage employers to hire a five per cent quota of disabled ex-servicemen. Historians have recently revisited the scheme, noting that in many cases the process was slow and fraught, with many disabled veterans facing the prospect of unemployment, yet few have paid attention to soldiers’ pre-war working backgrounds and the specific requests of British industries. This article focuses on British coalminers returning from war. What role was there in this national situation for an industry known for its own high rate of accident and injury? Although the King’s Roll made some attempt to find veterans specifically targeted jobs above and below ground according to their impairments, it proved unable to incorporate coalmining. Instead, many disabled ex-servicemen returned to the workplace and utilized their existing identities as miners to navigate the process. With the industry beginning to decline, many faced potential regression in job status, exploitation or unemployment. By shifting to an industry-specific focus, this case study explores the contested nature of work for disabled people after the First World War, and highlights the interrelation and importance of workplace identity for the returning disabled veteran.

At a special meeting in January 1919, the Miners’ Federation of Great Britain (MFGB) made a plea for the state to recognize the work situation facing disabled miners. Alongside demands for a reduction of the statutory working day from eight to six hours and the nationalization of the industry, the MFGB stated that discharged soldiers and sailors must be returned to their previous mines as soon as possible. In addition to this:

Demobilized men who are disabled, and unable to work in the mine, shall be trained for suitable occupations and paid during the training an allowance equal to what they would have earned in or about the mine had they not been disabled, that allowance to continue after training until suitable employment is found, the cost of training and allowance to be at the expense of the state.^1^

These demands were not met. All workers disabled after serving in the First World War returned home, forced to navigate not only the medical and social changes to their lives brought on by injury and disease, but a new and uncertain situation with regards to their employment. The primary work scheme available to British soldiers was the National Scheme for Disabled Ex-Servicemen, or the King’s Roll, planned in 1917 by the Manchester-based civil servant Henry Lesser Rothband and implemented in 1919. Employers who signed up to the scheme agreed to fulfil a quota of disabled ex-servicemen on their payroll.

Among the soldiers and sailors in the First World War were coalminers and recruits from coalmining communities who had already worked in an industry notorious for its high rates of death and injury. The extent of danger faced by miners is difficult to quantify fully, but Arthur McIvor and Robert Johnston estimate that ‘more than a quarter of a million miners were killed or permanently incapacitated as a result of injuries sustained whilst at their work over the period 1850–1950’.^2^ The death and mutilation of the First World War is equally impossible to comprehend. According to Joanna Bourke, seven per cent of all British men aged between fifteen and forty-nine were killed in battle.^3^ Added to this was the rapid rate of servicemen returning from the battlefield with permanent injuries. Meaghan Kowalsky notes that by February 1915 an average of 360 soldiers and sailors had returned home disabled each month.^4^ By 1922, an estimated 900,000 ex-servicemen were in receipt of a disability pension.^5^ The coal industry and the war acted as twin destructive forces in early twentieth-century Britain.

The employment situation for disabled veterans in the years after the First World War has been the subject of much discussion among historians, who have seen the British response variously as a largely successful programme of employment and as part of a broader picture of state failure for disabled veterans.^6^ However, this discussion is missing industry-specific analysis which explores the war’s effect on understandings of disability and work. What happened when it was impossible to return to an industry that provided not just an income, but also local and family connections and identity? The influx of returning soldiers into the mines sat uneasily with a work environment which was already responsible for huge amounts of industrial disease and that was already failing in many ways to incorporate disabled civilians. An approach that focuses in detail on industrial work helps us to look more closely at the economic and social situations affecting returning disabled ex-servicemen, which varied not just from industry to industry, but from person to person.

With this in mind, this article will explore the situation facing soldiers and sailors from British coalfield communities who returned home disabled from the war, and the struggle to return them to work around the pits. Many had been forced to stay in the industry due to its protected status, but this did not bar a significant proportion of miners from service: 313,000 of Britain’s 1.26 million miners had joined the forces by February 1916.^7^ The First World War and its aftermath came at a time of eminence and then rapid (though regionally varying) decline for Britain’s coal industry. This article will use examples drawn from three major British coalfields: South Wales, North-East England and Scotland. In South Wales and the North-East in particular local economies and communities were significantly based on coal, while the more spread-out coalfield areas of Scotland also provide comparative detail. These samples enable the construction of a national picture that also recognizes local variation within each area.

The post-war national effort to employ disabled ex-servicemen jarred with the implications of returning to an industry that was already known for its potential for death, disease and dismemberment. The approach used here helps to incorporate the figure of the disabled civilian into the picture, given that, as Courtney Andree has identified, the disabled civilian of the First World War has been almost completely neglected within existing historiography.^8^ The efforts of Whitehall to integrate disabled people into the coalmining workforce will be contrasted with the autonomously developing situation in the coalfields, in which production continued during the war and as disabled soldiers were brought back into the community. With the general failure of the King’s Roll to integrate coal into its provisions, disabled soldiers themselves navigated an employment situation that had major effects on their working and personal lives. A case study of the coal industry sheds light on the wider employment problems facing disabled veterans. Although many were re-employed, evaluation of the feelings of frustration, exploitation and political anger created by the failure in the coalfields helps to illuminate the wider importance of identity and space in the question of work for disabled people.

## Employing veterans: the National Scheme for Disabled Ex-Servicemen

Disabled soldiers and sailors returned from service to their previous homes with injuries and diseases from the war, unsure whether they could ever return to employment. Disabled veterans’ work quickly became a source for governmental discussion. The Ministry of Pensions set up an industrial training scheme which transferred to the Ministry of Labour in 1919. This provided work to disabled veterans, but experienced both limited take-up (fewer than 15 per cent of those eligible according to a 1917 estimate by the Secretary to the Ministry of Pensions) and limited availability. The most severely disabled ex-servicemen were rarely provided with substantial work, or had to face long delays.^9^ A 1922 pamphlet produced by the charity and activist group the Disabled Society, entitled *Handbook for the Limbless*, outlined this problem:

As the Government instructional factories were full, it was often months before a man could get a course, so he had to wait at home doing little or nothing. Such enforced idleness naturally did not improve his mental or physical condition, and made it harder for him to work when the opportunity arrived.^10^

No compulsory employment schemes were introduced by the state, in contrast to the war disability pensions administered by the newly formed Ministry of Pensions from 1916.^11^ Deborah Cohen contrasts this inadequate employment situation with the compulsory schemes in Germany, arguing that it ‘left the Ministry’s officials with little inclination to tackle the complicated problems that training programmes presented’.^12^ It thus fell to voluntary organizations to provide alternative schemes. Charities such as the Soldiers and Sailors’ Help Society and the Disabled Society set up workshops for training in handicrafts, or integrated work into wider programmes of medical rehabilitation.^13^

The National Scheme for Disabled Ex-Servicemen, or the King’s Roll, was the flagship governmental scheme for employing disabled veterans. The scheme emerged from a 1917 pamphlet authored by the Manchester rubber merchant Henry Lesser Rothband and entitled *Employment for Disabled Sailors and Soldiers: A Scheme for a National Roll of Employers*. Rothband’s system invited employers to pledge that five per cent of their employees were to be disabled ex-soldiers and sailors; in exchange, the employer would receive a place on the roll and use of the badge in publicity material and letterheads.^14^ Launched in September 1919, the scheme initially received a warm reception from the public and indeed employers, with 1452 companies signing up in the first week.^15^ By February 1920, 102,011 disabled ex-servicemen were employed under the scheme and 10,867 employers were using the badge.^16^ Yet the King’s Roll remained a voluntary scheme throughout its existence. The government denied any attempt to begin a full state employment programme, using the prestige of the badge and the contract of honour towards veterans as its mechanics, rather than state compulsion.

Although it is only relatively recently that historians have fully analysed the employment situation facing the First World War’s disabled veterans, the King’s Roll has come under scrutiny from multiple perspectives. Meaghan Kowalsky recognizes the historical significance of the scheme and also defends its success, arguing that it was not only successful in employing disabled soldiers but also in invoking a ‘wider debate regarding the employment of disabled ex-servicemen and the responsibility of the state, in part paving the way for future reforms’.^17^ Yet other historians have cast a more sceptical eye. Deborah Cohen is critical of the long-term effects of the King’s Roll and its failure to provide work specifically for more severely disabled people, arguing that ‘no profit-making venture could afford to hire or retain men whose disabilities had significantly reduced their productive capacity.’^18^

Indeed, it was soon realized that putting the most severely disabled veterans in a laissez-faire environment would not work. Some employers utilized disabled workers’ categorizations, assigning a percentage which indicated their disability according to ability to work. A severe impairment that prevented most kinds of work was classed as 100 per cent disabled, while 50 per cent disabled allowed for specifically tailored work.^19^ Not only did this reduce disabled people to numerical categorization: the fact that all came under one quota meant that employers were less likely to hire workers with higher percentages. The *Handbook for the Limbless* had made exactly this criticism of the scheme, arguing that it:

helps the disabled generally, but hardly the man who has lost an arm, for employers are inclined to take on the less than 50 per cent disabled. For this scheme a 20 per cent disabled man, and often less than 20 per cent, counts exactly the same as the 100 per cent.^20^

The remit of the King’s Roll covered all disabled men, regardless of level of disablement, but its organization did not make a specific effort to ensure severely disabled workers were given an equal opportunity to find suitable work. It was left to a minority of voluntary schemes to provide specific work for severely disabled veterans, such as the Poppy Factory set up in 1922 with the amalgamation of the Disabled Society and the British Legion.^21^ Criticism of the King’s Roll intensified and went hand in hand with broader anger among ex-servicemen about their unemployment, both disabled and non-disabled. The National Union of Ex-Servicemen was formed in 1919 with the protestation of disabled ex-servicemen’s unemployment at the heart of its agenda, and grew to almost 100,000 members in its first year.^22^

This failure to encompass the disabled population has led disability historians such as Anne Borsay to include the King’s Roll as part of broader patterns of post-war state failure towards disabled people.^23^ This is an important point, but the question of voluntary versus state response should not be the sole area of historical enquiry. The King’s Roll was one of several schemes and events which changed attitudes and experiences of work for disabled people immediately after the war. Looking at the King’s Roll with focus on a specific industry helps further problematize the situation by showing how the ideal of the disabled soldier ‘returning to work’ was complicated by the needs and demands of particular sectors of the industrial economy. The notion of returning to work was made significantly more difficult with an industry such as coal which, by the end of the First World War, had its own major issues with employing disabled people, and was on the cusp of an industrial crisis.

## The struggle to include coal

Although coal was designated as a protected industry, many miners went to war. An estimated twenty-five per cent of male coalminers were enlisted for military service in 1916.^24^ Some enlisted coalminers provided unique skills, such as the South Wales miners who formed the tunnelling companies on the Western Front which utilized their mining expertise.^25^ The British coal industry was effectively put under state control in 1915, when the Price of Coal (Limitation) Act limited coal price inflation and profit levels, leading essentially to the nationalization of coal.^26^ It was, however, hardly the vision of nationalization for which miners’ unions had been campaigning. Major strikes — technically illegal under the new legislation – became a presence in certain coalfields. Sixty-seven strikes were recorded in the industry in 1916, rising to 116 in 1917.^27^ The most notable of these was in South Wales where 200,000 miners staged industrial action over wages in July 1915.^28^ In addition to feelings of industrial unrest, the restricted industry saw a wartime decline in output, from 287.4 million tons at the start of the war to 227.2 million tons in 1918.^29^

As this suggests, the war also marked the end of the ‘comfortable superiority’ of the coal industry.^30^ Historians have largely pinpointed the start of the First World War as the end of its peak and the beginning of a decline. The year 1913 separates two major volumes about the history of the coal industry: Roy Church’s era of *Victorian Pre-eminence* between 1830 and 1913 and Barry Supple’s *Political Economy of Decline* from 1913 to 1946.^31^ Indeed, the inter-war years were largely a period of sharp decline in profit and prestige for the coal industry. This was, of course, a complex and regionally varied decline, affecting districts such as South Wales (heavily reliant on exports) more than the comparatively modernized and productive North Midlands coalfields.^32^ Yet the First World War remains a crucial turning-point for the industry, deserving of close scrutiny for its effects on coal. Disabled soldiers and sailors returning to the coalfields in need of work found themselves faced with the difficult notion of re-employment.

The King’s Roll aimed to include all major British industry, but coal presented specific problems from the outset. How would this dangerous industry fit into a programme of re-employment of disabled people? In September 1919, just over a week before the scheme commenced, the Minister of Labour outlined the problems facing any integration of coal into the scheme:

Owing to … the peculiar conditions of the [coal] industry, the very high percentage of industrial accidents, and the necessity for making provision for the employment of disability within the industry, the Ministry are satisfied that while the persons formerly employed within the Coal Mining Industry are not outside the purview of the scheme, there is little possibility of the Coal Mining Industry absorbing other than those who have previously worked in that Industry.^33^

The latter statement exposed the problems the industry already had in including disabled workers, which were long established.

Though a minority of miners returned underground after being permanently injured or contracting a disease, most were regularly offered ‘light work’ by their employers to replace their previous underground jobs. This often resulted if they had been working for a long time and had developed a relationship with the colliery and its owner. Medical referees often dictated the degree to which they were ‘fit for work’; thus surface work, picking tables and signalling were often offered to these workers if they could not work underground. For example, Charles Robertson, a Scottish miner with a strained back, was deemed by the refereeing doctor in the early 1910s to ‘never be fit for face work’ and ‘not fit to walk far underground’. Robertson was instead posted to work at the ‘picking tables’ sorting coal, while also receiving partial compensation.^34^ These jobs were often monotonous and came with a reduction in both pay and status, or were used to restrict miners’ access to disability compensation under the Employers’ Liability Act of 1880 or the Workmen’s Compensation Act of 1897.^35^

Coupling this continuous situation of disabled people’s work in the industry with an influx of disabled men needing limited work, the outlook was pessimistic. A transnational debate occurred at the second Inter-Allied Conference on the After-Care of Disabled Men, held in London in May 1918. Discussing soldiers with tuberculosis, Major P. Horton-Smith Hartley described mining as ‘clearly and obviously unsuitable to a consumptive patient’.^36^ Coal had already been designated an unsuitable industry for re-employment of disabled ex-servicemen. Thus when the first published list of companies on the King’s Roll appeared in 1920, a disclaimer was included which stated that:

The coal mining industry made arrangements to absorb into employment all disabled men who have previously worked in that industry. Owing to the very high percentage of industrial accidents, it was felt quite impossible to make arrangements to absorb other than disabled men who had previously been so employed.^37^

Coal shared this exception with shipbuilding and engineering, other dangerous industries with high accident rates.

Despite this, the Ministry of Labour did plan an attempt to integrate coal into its wider plans to employ disabled veterans. A 1919 Ministry of Labour document entitled ‘List of Occupations in Which Partially Disabled Men Might be Employed, if Otherwise Suitable’ attempted to match jobs to different types of impairment (see Table 1). These mining jobs were included among recommendations from many industries. The document divided colliery labour into jobs suitable for disabled veterans, with amputees recommended for either labouring or supporting jobs such as signalling, or surface work on the picking belts. No coal jobs were listed for ‘men who have lost two arms’, ‘men who have lost both feet or legs or use of same’, or ‘men suffering from blindness, total or partial’, again showing immediate doubt that severely disabled workers could be re-integrated at all. Despite this, the document showed a genuine ambition for the coal industry to work within the framework of the King’s Roll, though the inclusion of the general position of ‘coalminer’ under ‘men suffering from other infirmities but fit for light work’ is perhaps indicative of its uncertain and ambitious nature.

**Table 1.** Coalmining jobs in the Ministry of Labour’s ‘List of Occupations in Which Partially Disabled Men Might be Employed, if Otherwise Suitable’, 1919.

For men who have lost the use of one arm	Colliery Banksman, Mottyman (Collier’s Labourer), Colliery Banksman, Signalman in Coal Mine, Weighman
For men who have lost a foot or a leg or use thereof	Clipper-on (Colliery), Colliery Picking Belt Worker, Colliery Lamp Attendant, Weighman
For men suffering from other infirmities but fit for light work	Coal Miner

Source: TNA, LAB 2/1199/ED70510/1919, Ministry of Labour and Predecessors, correspondence concerning the decision to transfer the responsibility of analysing the disabilities of ex-servicemen in order to find them suitable employment, to the Ministry of Pensions (1919). It is notable that this document formed part of the general transition of responsibility for disabled veterans from the Ministry of Pensions to the Ministry of Labour.

The notion of retraining miners into a different industry was complicated by the social, geographical and allegedly physical ties that coalminers had to their work. Miners’ bodies had long been described in ways that emphasized miners’ physical difference from other workers, often in a racialized sense. Mining engineer James Barrowman, speaking to the Mining Institute of Scotland in June 1896, asserted that miners were ‘picked men’ and that ‘Those who are lame, blind and otherwise infirm, will not choose this occupation; and those who by accident are disabled for active exertion must leave it.’^38^ Thus war was seen to further disrupt an already questioned process of disabled bodies’ inclusion in the coalfield workforce. *The Times* in 1919 posed the question of disabled miners’ retraining, arguing that ‘difference of capacity and previous experience’ greatly affected the chances of re-employment and retraining. The article compared a ‘perfectly healthy’ miner to a clerk, the healthy miner being always ‘reasonably sure of employment’. However, ‘In disablement the advantage passes to the clerk, who possesses an aptitude that fits him for a dozen callings. … But the miner become a cripple cannot so easily adapt himself to sedentary existence; his hands require more discipline than the clerk’s mind.’^39^ The article presented the coal industry as unavailable to disabled people in a physical sense, in contrast to a secondary occupation for a clerk who could still utilize their ‘mind’, presenting an image of the coal industry as barred to disabled workers. Of course this ignored the fact that there was a disabled workforce within the mining industry. Nevertheless, it was clear that the limited places within the King’s Roll could hardly cater for those whose working lives had been tied to the coalfields.

As the King’s Roll scheme was being prepared and introduced, government officials, miners and coal-owners separately discussed the situation for disabled veterans. The Coal Controllers Advisory Board in August 1917 agreed to ‘find places for miners whom the Mining Industry could not, on the ground of the nature of their disability, receive back’, but with the understanding that ‘the Mining Industry should be regarded as self-contained’, with its own body investigating it.^40^ The coal-owners’ body, the Mining Association of Great Britain, met the MFGB for discussions about disabled ex-miners returning from the war. A conference in January 1918 discussed outdoor work, finding that there ‘was not much opportunity for open-air employment for such men in connection with the coal trade’.^41^ Yet some in Whitehall were finding the coal industry not just autonomous but actively hostile to government schemes. The Ministry of Pensions found that, in some cases, they were ‘meeting with difficulty … in obtaining from collieries information as to the previous earnings of disabled soldiers and sailors formerly miners’.^42^ It was clear that state welfare for disabled veterans was not going to cover the coalfields. Yet, while this was being discussed and delayed at policy level, disabled soldiers and sailors had begun returning to coalfield communities in need of work.

## Disabled soldiers returning to the coalfields

The link between soldiers and miners had been present in the coalfields long before the First World War. For one, the metaphorical comparison of soldiers and miners was a common feature of trade union arguments for miners’ rights. Ben Curtis and Steven Thompson identify a particularly resonant example from Vernon Hartshorn of the Maesteg District of the South Wales Miners’ Federation, who told the 1919 Coal Industry Commission that in ‘the mining industry the casualties are more like those of the battlefield than anything else’.^43^ The return of disabled veterans was an inevitable priority for mining communities and trade unions. Many did not return to work, and the general welfare of returning disabled soldiers and sailors to mining towns was pushed by their local unions. Some were successful in securing compensation for their war injuries on their return to the coalfields. For example, the Durham Miners’ Association (DMA) in 1918 dealt with scores of military disability cases, including disabled miners who had joined the army themselves, such as J. R. Batie, who had ‘ruptured himself’ at work on 28 January 1918 and joined the army eleven days later. When he returned, and after agreeing to an operation, the DMA secured full compensation from his discharge.^44^ Unions’ fight for disabled people’s rights encompassed their family lives as well; if men could not return to their previous jobs, then the focus would be on preserving the family lives that were inextricably tied to the mines. Thus in 1918, the South Wales Miners’ Federation’s Rhondda No. 1 District passed a motion ‘That this District take the necessary steps to enforce the Government to grant full allowance to all Children born to disabled Soldiers and Sailors after the date of their Discharge from Military or Naval Services.’^45^

Work was by no means widely available in the mines, and disabled ex-servicemen returning to the coalfields had to rely on multiple, often unreliable sources of welfare. Disabled miners in the early twentieth century were supposedly guaranteed compensation for injury by the 1897 Workmen’s Compensation Act, as well as a tapestry of voluntary sources which could include charity, friendly and mutual aid societies, trade unions and more informal family or community care. All of these were almost universally inadequate on their own, and both state and voluntary compensation schemes were often reluctant to provide welfare.^46^ Certainly, war disability pensions became part of the framework of welfare for miners. The formation of the Ministry of Pensions in 1916, along with a 1917 Royal Warrant and War Pensions Acts in 1919 and 1921, created a system of state pensions at odds with the anti-interventionism of state employment provision. Helen Bettinson has shown that the disabled claimants of these pensions had a degree of autonomy that has gone unrecognized by historians of welfare and disability.^47^ Yet many disabled miners struggled with the inadequacy of the networks of welfare available.

This fear can be seen in the Durham and Northumberland Coal Owners Association’s survey, sent to collieries in February 1919, which aimed to answer ‘the question of the housing of disabled soldiers or their widows and families’. The survey focused on widows and dependants of the deceased but also attempted to work out welfare expenditure for disabled ex-servicemen in the community.^48^ Almost certainly as a last resort, some disabled ex-soldiers and sailors turned to the workhouse for relief and medical care. According to the 1919 admissions book of the Merthyr Tydfil workhouse, three discharged soldiers with neurasthenia, gastritis and ‘giddiness’ were admitted to the workhouse infirmary, then discharged after a period ranging from three days to two weeks.^49^ Disabled ex-servicemen in coalfield communities had emerged quickly as an area of financial concern in policy, and were struggling to survive in practice.

A small minority of collieries provided work as part of the King’s Roll. The Glendinning Antimony Mines in Langolm, Dumfriesshire, was the only colliery listed in Scotland in the 1920 King’s Roll list.^50^ None were listed in North-East England, while two were in Wales: the Wernddu Railway & Colliery in Pontardawe and the Ebbw Vale Steel, Iron and Coal Co. In 1926, the MP for Abertillery, George Barker, described an example from the latter that he had seen in his constituency:

Passing my door in the last two or three years there has been a man employed by the Ebbw Vale Colliery Company who is without both arms. The company had a leather bag made for him and employed him in carrying messages from one office to another. I know for a fact that that company employed every ex-service man who was disabled, and I have great pleasure of testifying to that on the Floor of the House.^51^

This was indeed a successful use of the King’s Roll by a colliery company, but clearly came with a major change in the experience of work for the miner. It was also hardly representative: the Ebbw Vale company had been one of a few firms to sign up officially for a position on the Kings’ Roll, probably due to its links with the steel company, and much like the Wernddu’s joint status as railway and colliery.

Instead, almost all coalminers who did return to the mines did so within the structures of the companies rather than through the auspices of the state. Some disabled ex-servicemen returned directly to their old mines to resume their jobs. An example was Haydn Thomas from Bedlinog, South Wales, who was called up to fight in 1916, and returned home having been wounded. According to his 1974 interview, he returned to a haulage job after the war.^52^ Returning to work in a physically demanding industry proved a major challenge for disabled ex-coalminers. In a 1997 interview the Scottish miner Robert Hamilton remembered his father returning from the First World War having contracted tuberculosis. He returned to the pit as a haulage worker ‘but he wisnae long, only a few weeks working when he collapsed. And had to be carried up the pit and he never went back again after that.’^53^ A return to the coalface also brought with it a return to the dangers of mining work. Miner George Hitchin from Seaham, County Durham, wrote about his uncles in his 1962 memoir *Pit-yacker*, one of whom ‘lost his right leg at Ypres (and his left some years later in a pit accident)’, an example of both war injuries and mining accidents occurring and separately changing men’s experience of work.^54^

 The difficulties in accommodating disabled soldiers meant limited work availability for the *entire* disabled population in the coalfields, including those who had not gone to fight. In South Wales this became a particular source of tension. In January 1920, John Harris, a worker in Dowlais who contracted the eye condition miners’ nystagmus, was told by a Surface Official ‘that [the Official] had given Harris’ work to another workman – a Discharged Soldier’. Harris had previously been receiving full compensation and had returned underground two years earlier, but stopped working as a result of nystagmus and the 1919 coal strike.^55^ Although unclear whether the soldier had also been discharged for disability, Harris’s realization that his job had been given away demonstrates the tight circumstances for employing disabled workers. It also reflects Joanna Bourke’s assertion that, in a squeezed labour market for disabled people, disabled civilians ‘fell to the bottom of the heap’.^56^

However, this was not a simple case of a hierarchy between disabled war veterans and disabled civilians. The tension between the two was a matter of regional difference, as recognized by Kowalsky who notes that work for disabled ex-servicemen was particularly difficult to find in south Wales and the north of England because so much was centred on labouring.^57^ The attitudes of these employers varied by region and by colliery. This was hinted at by John Henry Palin, the MP for Newcastle-upon-Tyne West who, in a 1926 debate about the King’s Roll, compared Yorkshire to his constituency, where ‘the type of employer, I regret to say, is not so generous’. He invoked a hypothetical civilian miner ‘who has been demobilized all this time, and who appeals very pathetically either for employment or training to fit him for some job where he can get his living independent of charity’.^58^ This lack of jobs and element of competition was recognized by the unions as having potential for further tension or even open exploitation. In 1918, a year before the start of the King’s Roll, coal-owners in Rhondda were scorned by the local branch of the South Wales Miners’ Federation for:

employing Men who have been Disabled in Warfare at Reduced Rates, thus using these Men, whose power to Labour has been minimized by the destructive force of Militarism, to cut down present rates which have been won after great efforts by the Miners’ Federation.^59^

This covert manipulation of the labour of ex-servicemen thus became a further area of contention in a volatile environment of labour relations. War in this model had an economically disabling effect, reducing the ‘power to Labour’ in a way that could be exploited. It suggests a complex relationship between disabled veterans within an industry which ostensibly prioritized them as workers, but in a way that could be unwanted, exclusive and even exploitative.

Many disabled soldiers and sailors returned from the war having to start completely new training. It was widely recognized that, although preferable, it was not always possible to return to a previous occupation. The *Handbook for the Limbless* noted that ‘the choice of occupation … largely depends on the mental and physical disability and on previous experience’. Although ‘it is much better for a man to return to his former trade’, many had the time-consuming and potentially stressful experience of learning a new trade from the beginning.^60^ This was the case for many miners. In 1922, MP for Renfrewshire Eastern Joseph Johnstone – who also acted as the chairman of the local Technical Advisory Committee in charge of retraining veterans – recounted the story of a miner in his constituency who was retrained in one of the government factories especially for this purpose:

On the occasion of one of my visits to a training factory, I saw a young lad who had only one arm. He was engaged at cabinet work, and was making some very beautiful tea trays, and was making a very good job of it, I asked him what he had been before he went into the Army, and he said that he had been a coalminer. When he came before the Technical Advisory Committee they asked him what he would like to do. He had a notion of joinery work. He felt he had a bent in that direction. They put him to that work. In a very few weeks he made very good progress. That is the difficulty of local Technical Advisory Committees. They have got before them a class of people who have to choose some form of employment in which they were not engaged before, and they have to put them to the occupation for which they think them best fitted. To that extent there is as much elasticity in the scheme as could be expected.^61^

The ex-miner appeared to be doing well in the new job, but Johnstone’s somewhat resigned tone demonstrates his recognition of the displacement of identity of what was termed an entire ‘class of people’, from working as a miner in the coalfields to doing something completely new.

Contemporary literature offers a window into the impact of this radical alteration of work on disabled miners and their working identities, families and relationships. Coalfield novels featuring characters disabled from war highlight a sense of injustice at the lack of work offered upon their return. A. J. Cronin’s *The Stars Look Down*, set in a mining community in North-East England, features a miner and war veteran ‘lame’ from a shot in the leg, who is refused work when he returns to the colliery. As he queues for the dole, he delivers an angry speech:

We was the nation’s f— heroes, and what are we now? Shiftless lazy scum. … We was told to fight for England – our own beloved soil. Christ! We fought for it, didden’t we? And we’ve f— well got it. We’re standing in it now. And what is it? Muck! Plain muck! But ye cannot eat muck, lads.^62^

The novel, based on Cronin’s experiences as a doctor working with miners, hinted at a loss felt by miners that was both emotional and financial.

The identity that had been damaged by unemployment was an unquestionably masculine one. The theme appeared in the work of the Welsh novelist Jack Jones. His brother Dick returned from the war ‘unable to find the light work [in the colliery], which was all he was capable of owing to his shattered hip’.^63^ His novel *Black Parade* featured the character Lewis, clearly based on his brother, who had a shattered hip and organ damage after the First World War. Lewis describes the stoppage of 1921 as placing his father and ‘most of the miners of his generation, the used-up generation’, on the retired list.^64^ The novel contrasts their unemployment with the other disabled members of the family: Benny (who lost an arm in the First World War) who sells insurance, Ossie (weaving baskets at the blind institution) and Lewis himself, a book-keeper. Jones’s characters illustrate a declining industry and changing expectations for disabled people’s work while still taking place within a family structure. Wendy Gagen has written of this as part of a social and emotional construction of disability: though early experiences of disability after war were ‘exacerbated by the experience of pain and the gendered codes that surrounded it’, workers’ gender identity was not invalidated, creating ‘new visions of selfhood that nevertheless often adhered to masculine ideals’.^65^ Nevertheless, this new conception of work had unwillingly moved away from the space of the coalmine.

## Reviewing the situation: the 1922 Select Committee on Training and Employment of Disabled Ex-Servicemen

Within three years of the implementation of the King’s Roll, initial frustrations regarding disabled employment gave way to harsh criticism. The Select Committee on Training and Employment of Disabled Ex-Servicemen was set up to investigate the status of the voluntary systems of employment training. The Committee’s report in 1922 reviewed the first three years of the King’s Roll, noting that its numbers were declining and many quota contracts had not been renewed, while an estimated 10,000 disabled ex-servicemen were unemployed. ‘The voluntary system’, warned the report, ‘is failing.’^66^ Interviews with those involved in returning disabled ex-servicemen to work in mining communities expressed their anger at the failures of the scheme. In particular, it was identified that the scheme was not catering for people with more severe physical disabilities, who were given little recognition of the extra difficulties they faced in getting work. David Watts Morgan, MP for Rhondda, told the Committee the ‘chief difficulty’ in his district was ‘men who have broken down who have gone past the period when they can claim that they are entitled to get training … they are now scrapped to the world, and they have nobody to help them’.^67^ Morgan’s response was a particularly emotional one amid many complaints to the Select Committee about the lack of work and training for miners.

A question session with head of the Committee, Montague Barlow, MP for Salford South, revealed the vulnerability of mining jobs for disabled ex-servicemen, subject as they were to other economic conditions which were being felt in the post-war years. These included the 1921 coal strikes and the declining trade of the coal industry. Watts Morgan posited this situation, which he was familiar with in his constituency:

A man is in receipt, shall we say, of a 20 per cent pension. He is disabled, and has gone back to his employer and said to him: ‘I have fought for my country, and you made me a promise to take me back.’ The employer says, ‘Of course, I did make you a promise to take you back; I will keep it.’ But the colliery closes, and the man with a 20 per cent disability is on the market. He is not efficient, and cannot go anywhere else, except to the workhouse – which you are telling him to do?^68^

Barlow argued that ex-servicemen like this would qualify for the scheme, but recognized that ‘that man, being a disabled man … is not, of course, as efficient as an able-bodied man’.^69^ Disability was portrayed as an economic process, the disabled miner reduced to terms of measurable efficiency. Pit closure was a particular issue. Another questioner of Barlow, Jack Lawson, put such a situation in blunter terms: ‘Apart from the special goodwill of that employer who is now compelled, say, to close his mine or something like that, that man does not stand a dog’s chance of getting employment even as a labourer.’^70^ The exchange served as an admission that the government’s scheme was not able to cater for the full extent of disability in the coalfields. It also brought to light what Morgan termed those who ‘cannot go anywhere else’. Their lives – their work, their home and their personal identities – were tied to the coalfields.

The dissatisfaction with the King’s Roll in the coalfields – manifested in the House of Commons as political anger – worsened over time. A few months before the General Strike and the miners’ lockout, in February 1926, MPs once again attacked the King’s Roll for its failure to integrate coalmining. John Henry Palin of Newcastle summarized the toxic combination of poor economic conditions and reluctant colliery owners that created a situation where ‘there are a great many disabled miners for whom the mining companies seem to have very little sympathy’ and called on either a compulsory scheme or another way of compelling ‘some very big firms who have taken up a totally irreconcilable attitude to this question’.^71^ Two years later, another Commons debate found Duncan Graham, MP for the Hamilton area of Lanarkshire, Scotland, criticizing coal-owners who were not co-operating with the King’s Roll pledges, which was met by an apologetic declaration that ‘regards must be had to the fact that the industry finds employment for a number of men who have been disabled while working in the mines’.^72^ The rest, however, felt let down both by employers and the state. Later in his speech, Palin attempted to salvage the reputation of employment schemes among the coalmines: ‘I feel that, generally speaking, Government contractors and public authorities have already done all that it is possible for them to do in this direction.’ Met with a chorus of ‘No!’ he decided that ‘we shall have to leave that a moot point.’^73^

## Conclusion

The King’s Roll continued to operate throughout the inter-war years – as did other voluntary schemes such as local training workshops – with continuous public support but increasing problems and high inter-war unemployment.^74^ Yet the problems posed by the coal industry, which had been recognized as problematic even before the King’s Roll began, were never solved. This article has argued that the national situation regarding disabled ex-servicemen’s employment should be understood in the context of British industry. Looking at the issue of returning disabled ex-servicemen from a single-industry perspective offers invaluable detail, illuminating problems which have not yet been sufficiently explored by historians. The coal industry’s economic and political tensions formed a complex social background to the returning coalminer, and directly affected the kinds of provisions available to them. The organizers of the King’s Roll made an earnest attempt to include coal in its workings, communicating with coal-owners and including some jobs in and around the colliery. However, the scheme failed to integrate disabled soldiers into what was already a dangerous and declining industry. The system also failed to encompass the spectrum of impairment, admitting the exclusion of the most severely disabled veterans. Those disabled ex-servicemen who were given ‘light’ service employment experienced major changes to their working identities and personal lives, including their family relationships and gender identity, which were inextricably linked to the coalfields.

Disabled soldiers returning to the coalfields instead exercised autonomy and used their existing connections with employers and unions in securing further work or other modes of welfare, including compensation. However, unemployment among disabled ex-servicemen was widespread, and caused many disabled ex-servicemen to have either their employment or welfare needs ignored. Added to this, the scarcity of employment led ex-servicemen and disabled civilians to be compared as to who could be the most economically ‘efficient’. Individual employers and workers experienced wildly varying situations in this regard. ‘Light work’ was in some cases prioritized for disabled ex-servicemen, but veterans could also be particularly exploited as cheap labour. Coal was a particularly resonant example of an industry that could not operate within the official structures of post-First World War employment, but it was not the only one. Engineering and shipbuilding were also identified along with coalmining as problematic early in the development of the King’s Roll, and both deserve further historical study. The picture of state failure and employment uncertainty in the years after the First World War preceded a shift away from mining employment to spaces outside the coalfield. The process was to intensify later with the passing of the Disabled Persons (Employment) Act in 1944, which legislated for vocational training and industrial rehabilitation courses for disabled veterans, and finally made quotas of disabled workers compulsory.^75^ After the Second World War, Remploy factories, which provided factory employment to severely disabled workers, were to bring this style of sheltered work directly to coalfield areas across Britain.^76^ The disabled veterans of the First World War set a precedent for this shift, facing an even more uncertain future as they returned to the coalfields.

## Funding

This work was supported by the Wellcome Trust [Grant no. 095948/Z/11/Z].

## Disclosure statement

No potential conflict of interest was reported by the author.

## Footnotes

^1^ The Times, 16 January 1919, 3.

^2^ A. J. McIvor and R. Johnston, *Miners’ Lung: A History of Dust Disease in British Coal Mining* (Aldershot, 2007), 42.

^3^ J. Bourke, *Dismembering the Male: Men’s Bodies, Britain and the Great War* (London, 1999), 15.

^4^ M. Kowalsky, ‘ “This honourable obligation”: the King’s National Roll Scheme for Disabled Ex-Servicemen 1915–1944’, *European Review of History: Revue Européenne d’histoire*, 14, 4 (2007), 567–84, 568.

^5^ BPP, *Report from the SC on Training and Employment of Disabled Ex-Servicemen*, 1922 (170), 1.

^6^ D. Cohen, *The War Come Home: Disabled Veterans in Britain and Germany, 1914–1939* (Berkeley, 2001); Kowalsky *op*. *cit*.; A. Borsay, *Disability and Social Policy in Britain since 1750: A History of Exclusion* (Basingstoke, 2005); Bourke, *op*. *cit*.

^7^ J. M. Winter, ‘Britain’s “Lost Generation” of the First World War’, *Population Studies*, 31, 3 (1977), 449–66, 452.

^8^ C. Andree, ‘Non-combatants and other peace activists: “everyday” disability in a time of war’, *Disability Studies Quarterly*, 34, 3 (2014); available online at: http://dsq-sds.org/article/view/3773 (accessed 7 January 2015).

^9^ J. Anderson, *War, Disability and Rehabilitation in Britain: ‘Soul of a Nation’* (Manchester, 2011), 46–8.

^10^ G. Howson, *Handbook for the Limbless* (London, 1922), 68.

^11^ The long delay and chaotic administration of the pension scheme was subject to intense criticism, including a volatile demonstration in May 1919 in Hyde Park, London, by disabled veterans and their supporters. Cohen, *op*. *cit*.; H. Bettinson, ‘ “Lost Souls in the House of Restoration”? British Ex-Servicemen and War Disability Pensions, 1914–1930’ (Ph.D., University of East Anglia, 2002).

^12^ Cohen, *op*. *cit*., 26.

^13^ Anderson, *op*. *cit*., 44.

^14^ For a full account of the foundation of the scheme, see Kowalsky, *op*. *cit*. Rothband’s was not the only scheme being prepared. For example, Thomas Young of the Edinburgh and East of Scotland College of Architecture outlined a scheme for disabled soldiers to move to rural locations and train in occupations such as ‘golf club making’ and ‘forest nursery’, with the wider aim of reviving rural occupations and the ‘regeneration of village life’: T. Young, *Discharged and Disabled Sailors and Soldiers: Suggested scheme for the training and employment of discharged and disabled sailors and soldiers* (Glasgow, 1917).

^15^ Kowalsky, *op*. *cit*., 571.

^16^ Howson, *op*. *cit*., 95.

^17^ Kowalsky, *op*. *cit*.; Kowalsky’s contribution to the field is also discussed in the introduction to the 2007 issue of *European Review of History* in which her essay appeared. See J. Anderson and A. Carden-Coyne, ‘Enabling the past: new perspectives in the history of disability’, *European Review of History*, 14, 4 (December 2007), 447–57.

^18^ Cohen, *op*. *cit*., 40.

^19^ Anderson, *op*. *cit*., 47.

^20^ Howson, *op*. *cit*., 69.

^21^ Anderson, *op*. *cit*., 47.

^22^ E. Elsey, ‘The Rehabilitation and Employment of Disabled Ex-Servicemen after the Two World Wars’ (Ph.D., University of Teesside, 1994), 42. Elsey notes the organization’s ‘far-sighted’ approach in involving widows, wives and families of ex-servicemen as well as women who had served in the medical services.

^23^ Borsay, *op*. *cit*., 128.

^24^ W. Notz, ‘The world’s coal situation during the war: I’, *Journal of Political Economy*, 26 (1918), 567–611. J. M. Winter argued that the lasting image of the ‘Lost Generation’ of men killed in war has focused disproportionately on elites and has overlooked working-class recruits, particularly miners: Winter, *op*. *cit*., 449–66.

^25^ R. E. Wood, ‘The South Wales Miners’ Contribution to the Tunnelling Companies on the Western Front during the Great War’ (Ph.D., Swansea University, 2012).

^26^ J. M. Bourne, *Britain and the Great War 1914–1918* (London, 1989), 194.

^27^ Notz, *op*. *cit.*, 596.

^28^ N. Whiteside, ‘The British population at war’ in John Turner (ed.), *Britain and the First World War* (London, 1988), 94.

^29^ P. Howlett and S. Broadberry, ‘The United Kingdom during world war: business as usual?’ in Stephen Broadberry and Mark Harrison (eds), *The Economics of World War I* (Cambridge, 2005), 213.

^30^ N. K. Buxton, *The Economic Development of the British Coal Industry from Industrial Revolution to the Present Day* (London, 1978), 98.

^31^ R. A. Church, *The History of the British Coal Industry Volume 3: 1830–1913: Victorian Pre-eminence* (Oxford, 1986); B. Supple, *The History of the British Coal Industry Volume 4: 1913–1946: The Political Economy of Decline* (Oxford, 1987).

^32^ Supple, *op*. *cit*., 3; D. Greasley, ‘Fifty years of coal-mining productivity: the record of the British coal industry before 1939’, *Journal of Economic History*, 50, 4 (December 1990), 877–902, 880.

^33^ The National Archives (subsequently TNA), LAB/2/1199/EDX433/1919, Ministry of Labour and Predecessors: correspondence with Employment Exchange Managers, Local Advisory Committees, Federations, Trade Unions and employers associated with the shipbuilding, engineering and coalmining industries. Regards the National Scheme for Disabled Ex-Service Men (1919).

^34^ National Archives of Scotland (subsequently NAS), CB 19/1, 2056/12, Scottish Coal Workers’ Compensation Scheme, Directors’ Minute Books, 1912–1914, Charles Robertson *v*. The Fife Coal Co. Ltd.

^35^ For the history of these compensation laws, see P. W. J. Bartrip, *Wounded Soldiers of Industry : Industrial Compensation Policy 1833–1897* (Oxford, 1983); P. W. J. Bartrip, *Workmen’s Compensation in Twentieth Century Britain : Law, History and Social Policy* (Aldershot, 1987).

^36^
*The Inter-Allied Conference on the After-Care of Disabled Men* (London, 1918), 271.

^37^
*The King’s National Roll* (London, 1920), xi.

^38^ J. Barrowman, ‘The health conditions of coal mining’, 13 June 1896; available online at: http://www.scottishmining.co.uk/430.html (accessed 7 January 2015).

^39^
*The Times*, 11 February 1919, 4. Implied in the notion of a ‘sedentary existence’ was the ability to use assistive mobility technologies at work, seemingly unavailable in the mining industry. This was, in fact, a source of great innovation within coal, with miners’ own health and welfare organizers leading provision of artificial limbs and assistive technology. See, for example, B. Curtis and S. Thompson, ‘ “A plentiful crop of cripples made by all this progress”: disability, artificial limbs and working-class mutualism in the South Wales Coalfield, 1890–1948’, *Social History of Medicine*, 27, 4 (2014), 708–27.

^40^ TNA, BT 189/1, Board of Trade: Coal Controllers Advisory Board – Notes of Meetings, 1917–1918, 22 August 1917.

^41^ TNA, BT 189/1, Board of Trade: Coal Controllers Advisory Board – Notes of Meetings, 1917–1918, 9 January 1918.

^42^ TNA, BT 189/1, Board of Trade: Coal Controllers Advisory Board – Notes of Meetings, 1917–1918, 30 January 1918.

^43^ BPP, *Coal Industry Commission*, vol. I: Reports and Minutes of Evidence on the first stage of the inquiry, 1919 (Cmd 359), I, 362; Curtis and Thompson, *op*. *cit*., 708.

^44^ Durham County Record Office (subsequently DCRO), D/DMA (Acc: 1004(D)), 167, Durham Miners’ Association, Compensation Department monthly reports, September 1918–December 1919.

^45^ Swansea University, South Wales Coalfield Collection (subsequently SWCC), MNA/NUM/3/8/10a, South Wales Miners’ Federation, Rhondda No. 1 District, Monthly Reports, 1901–1931, 11 March 1918.

^46^ For examples of this mixed economy of welfare, see Bartrip, *op. cit*. (1983); Bartrip, *op. cit*. (1987).; S. Cordery, *British Friendly Societies, 1750–1914* (Basingstoke, 2003); J. Benson, ‘Coalminers, coalowners and collaboration: the miners’ permanent relief fund movement in England, 1860–1895’, *Labour History Review*, 58, 2 (August 2003), 181–94; Curtis and Thompson, *op*. *cit*.; S. Thompson, ‘The friendly and welfare provision of British trade unions: a case study of the South Wales Miners’ Federation’, *Labour History Review*, 77, 2 (2012), 189–210; McIvor and Johnston, *op*. *cit*.

^47^ Bettinson, *op*. *cit*.

^48^ DCRO, NCB I/CO 876/762, Disabled Soldiers and Sailors or their Widows and Families, Housing Of, February 1919.

^49^ Glamorgan Archives (subsequently GA), UM/30/12, Merthyr Tydfil Union workhouse admission register, 1916–1921. The role of the Poor Law for miners is an under-studied area. John Benson has addressed this gap in the nineteenth century, arguing that ‘there has been virtually no interest … in the provision that the mid- to late Victorian state made – or failed to make – for its many citizens who became victims of industrial accidents’: J. Benson, ‘Poor Law guardians, coalminers, and friendly societies in northern England, 1860–1894: statutory provision, local autonomy, and individual responsibility’, *Northern History*, 34 (2007), 159–68, 159.

^50^
*The King’s National Roll* (London, 1920).

^51^ BPP, HC Deb, vol. 191 cc1829–65, 16 February 1926. Barker’s assertion that, ‘Personally I would rather see the Government responsible for employing disabled ex-service men than place the obligation on any private firm or individual’ demonstrates his stance against the hands-off stance of the government towards providing employment to veterans.

^52^ Swansea University, South Wales Miners’ Library, AUD/224, Haydn Thomas oral history interview by Alun Morgan (April 1974).

^53^ Strathclyde University, Scottish Oral History Centre Archive, Coal Mining Oral History Project, Robert Hamilton interview by Ian MacDougall, 9 June 1997.

^54^ G. Hitchin, *Pit-yacker* (London, 1962), 14.

^55^ GA, DXHV/35, S. O. Davies Papers, detailed list of members, with particulars of claims and compensations, 30 January 1920.

^56^ Bourke, *op*. *cit*., 55.

^57^ Kowalsky, *op*. *cit*., 570.

^58^ BPP, HC Deb, vol. 191 cc1829–65, 16 February 1926.

^59^ SWCC, SWCC/MNA/NUM/3/8/10a, South Wales Miners’ Federation Rhondda No. 1 District, Monthly Reports, 1901–1931, 8 April 1918.

^60^ Howson, *op*. *cit*., 97.

^61^ BPP, HC Deb, vol. 153 cc656–99, 26 April 1922.

^62^ A. J. Cronin, *The Stars Look Down* (London, 1935), 498.

^63^ J. Jones, *Unfinished Journey* (Oxford, 1937), 190.

^64^ J. Jones, *Black Parade* (Cardigan, 2009[1935]), 334*.*

^65^ W. J. Gagen, ‘Remastering the body, renegotiating gender: physical disability and masculinity during the First World War, the case of J. B. Middlebrook’, *European Review of History*, 14 (2007), 525–41.

^66^ BPP, *Report from the SC on Training and Employment of Disabled Ex-Servicemen*, 1922 (170), 1.

^67^ BPP, *Report from the SC on Training and Employment of Disabled Ex-Servicemen*, 1922 (170), Minutes of Evidence, 29.

^68^
*ibid*., 289.

^69^
*ibid*.

^70^
*ibid*., 288.

^71^ BPP, HC Deb, vol. 191 cc1829–65, 16 February 1926.

^72^ BPP HC Deb, vol. 219 c2472W2472W, 12 July 1928.

^73^ BPP, HC Deb, vol. 191 cc1829–65, 16 February 1926.

^74^ Cohen, *op*. *cit*., 45–6.

^75^ Disabled Persons (Employment) Act, 1944. For discussion of this Act see Borsay, *op*. *cit*.; Kowalsky, *op*. *cit*.; R. Cooter, *Surgery and Society in Peace and War: Orthopaedics and the Organization of Modern Medicine, 1880–1948* (Basingstoke, 1993), 213.

^76^ J. L. Edwards, ‘Remploy: an experiment in sheltered employment for the severely disabled in Great Britain’, *International Labour Review*, 77 (1958), 147; V. Long, ‘Rethinking post-war mental health care: industrial therapy and the chronic mental patient in Britain’, *Social History of Medicine*, 26, 4 (2013), 738–58; Elsey, *op*. *cit*., 276.

